# Case Report: Perifoveal exudative vascular anomalous complex with secondary fundus hemorrhage

**DOI:** 10.3389/fmed.2026.1828411

**Published:** 2026-06-04

**Authors:** Ziqing Liu, Xiaoyan Zhang, Xiujuan Du, Chunhuan Niu, Fang Sha, Bo Wen, Yane Gao, Ying Wen

**Affiliations:** 1 Affiliated Eye Hospital of Shandong University of Traditional Chinese Medicine, Jinan, China; 2 Shandong Provincial Key Laboratory of Integrated Traditional Chinese and Western Medicine for Prevention and Therapy of Ocular Diseases, Jinan, Shandong, China; 3 Shandong Academy of Eye Disease Prevention and Therapy, Jinan, China; 4 Ophthalmology and Optometry Medical School, Shandong University of Traditional Chinese Medicine, Jinan, China

**Keywords:** fundus fluorescein angiography, indocyanine green angiography, intravitreal anti-VEGF therapy, optical coherence tomography, optical coherence tomography angiography, perifoveal exudative vascular anomalous complex, PEVAC, secondary fundus hemorrhage

## Abstract

**Background:**

Perifoveal exudative vascular anomalous complex (PEVAC) is a rare macular disorder characterized by isolated, circular aneurysmal lesions surrounding the fovea centralis, typically occurring in otherwise healthy patients without retinal inflammation or vascular abnormalities. This study aimed to highlight a rare case of PEVAC with secondary fundus hemorrhage.

**Case presentation:**

This study reports the case of a 44-year-old Chinese woman with PEVAC complicated by secondary fundus hemorrhage. The PEVAC diagnosis was confirmed through fundus fluorescein angiography, indocyanine green angiography, optical coherence tomography, and optical coherence tomography angiography. After intravitreal anti-vascular endothelial growth factor (anti-VEGF) treatment, the patient experienced marked improvement in visual acuity, reaching 20/20.

**Conclusion:**

The case presented in this study underscores that PEVAC may lead to the rupture and hemorrhage of secondary lesions. Gradual improvement in the patient’s vision was achieved through intravitreal anti-VEGF injections. This study found that the menstrual cycle in women may influence disease progression. However, the relationship between the menstrual cycle and disease progression remains unclear.

## 1 Introduction

Perifoveal exudative vascular anomalous complex (PEVAC) is a rare macular disorder first described by Querques et al. (1) in 2011. PEVAC presents clinically as isolated, circular aneurysmal lesions surrounding the fovea centralis, typically occurring in otherwise healthy patients without retinal inflammation or vascular abnormalities. However, its pathogenesis remains unclear. PEVAC with secondary fundus hemorrhage presents a unique clinical scenario that warrants thorough examination and discussion. This study used intravitreal anti-vascular endothelial growth factor (anti-VEGF) therapy for PEVAC. This case report offers clinical insights into the therapeutic management of this condition.

## 2 Case report

**Figure 1:**
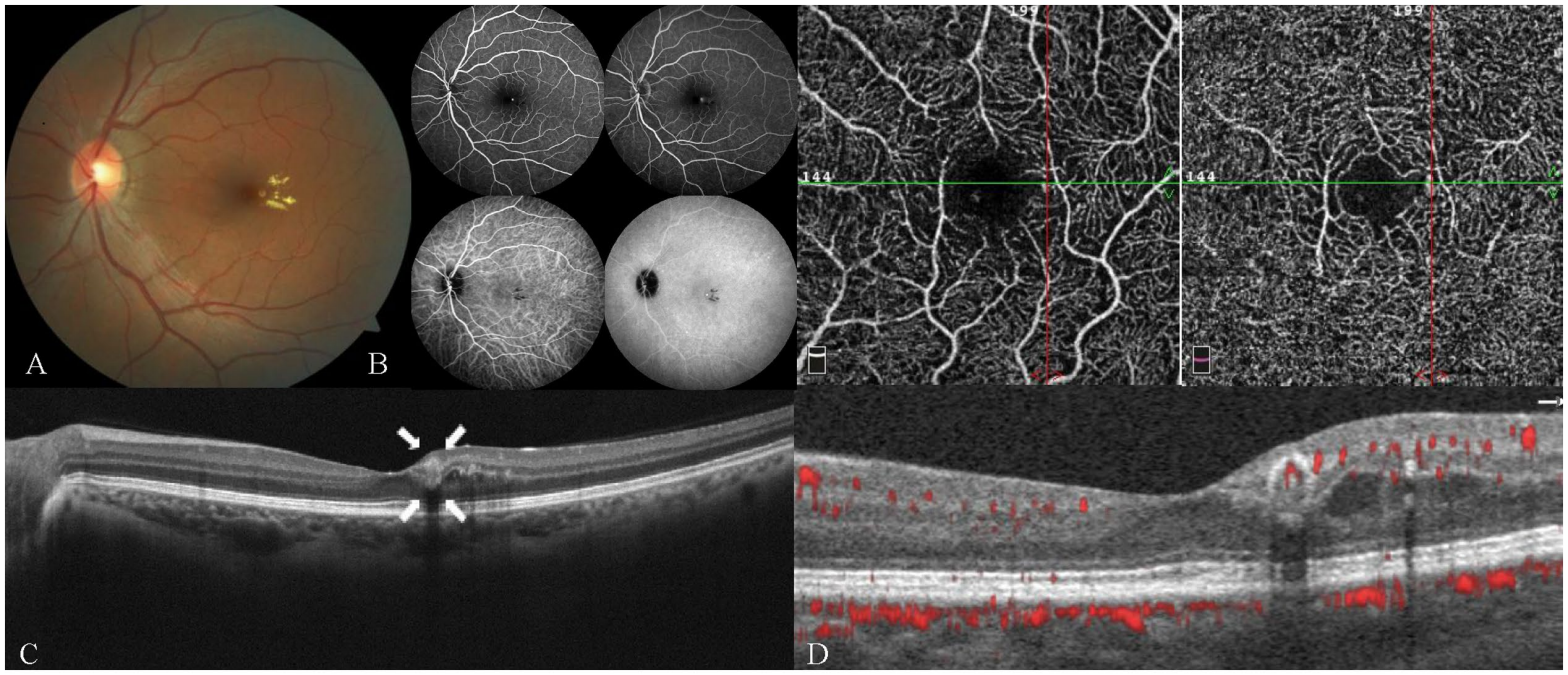
Fundus photograph of the left eye shows an isolated, circular aneurysmal lesion adjacent to the fovea centralis, surrounded by scattered yellow-white hard exudates **(A)**. FFA revealed a hyperfluorescent circular lesion in the arcuate temporal region of the left macula, with mild leakage developing as the contrast time progressed; ICGA showed no leakage in the late phase of the same hyperfluorescent lesion **(B)**. OCT revealed a round cystic lesion adjacent to the fovea in the left eye. The cyst wall exhibited higher reflectivity than the cyst cavity, with surrounding retinal interlamellar cystic edema and punctate hyper-reflective signals **(C)**. OCTA revealed an isolated arcuate vascular lesion in the temporal region of the left macula, with abnormal blood flow signals within it and no abnormal changes in surrounding capillaries. The abnormal blood flow signals were visible both in the superficial and deep capillary layers of the lesion **(D)**.

A 44-year-old Chinese woman presented to an outpatient clinic on 16 November 2023, with a 3-week history of decreased vision in her left eye. The patient had a history of thyroid nodules that had been under clinical observation without medication. The patient denied having diabetes, hypertension, or a family history of genetic disorders. A general physical examination revealed no abnormalities. The ophthalmologic examination recorded visual acuity findings as follows: right eye 20/80 corrected to 20/20 (−1.25/−0.50 * 69) and left eye 20/63 corrected to 20/50 (−0.75/−0.25 * 121), with normal intraocular pressure bilaterally (right eye 17.7 mmHg, left eye 19.7 mmHg). No significant abnormalities were observed during anterior segment examination. Fundus examination of the right eye showed no abnormalities, and the optic disc margin of the left eye was clear and normal. However, an isolated, circular aneurysmal lesion was visible adjacent to the fovea centralis, surrounded by scattered yellow-white hard exudates (Figure 1A). Fundus fluorescein angiography (FFA) demonstrated a hyperfluorescent circular lesion in the arcuate temporal region of the left macula, with mild leakage developing as the contrast time progressed. Indocyanine green angiography (ICGA) showed no leakage in the late phase of the same hyperfluorescent lesion (Figure 1B). Optical coherence tomography (OCT) revealed a round cystic lesion adjacent to the fovea in the left eye. The cyst wall exhibited higher reflectivity than the cyst cavity, with surrounding retinal interlamellar cystic edema and punctate hyper-reflective signals (Figure 1C). The lesion extended from the retinal nerve fiber layer to the outer nuclear layer of the retina, with diameters of 199 μm and 159 μm, respectively. OCT angiography (OCTA) revealed an isolated arcuate vascular lesion in the temporal region of the left macula, with abnormal blood flow signals and no abnormal changes in the surrounding capillaries. Abnormal blood flow signals were visible in both the superficial and deep capillary layers of the lesion (Figure 1D). At this time, the diagnosis was PEVAC in the left eye.

**Figure 2:**
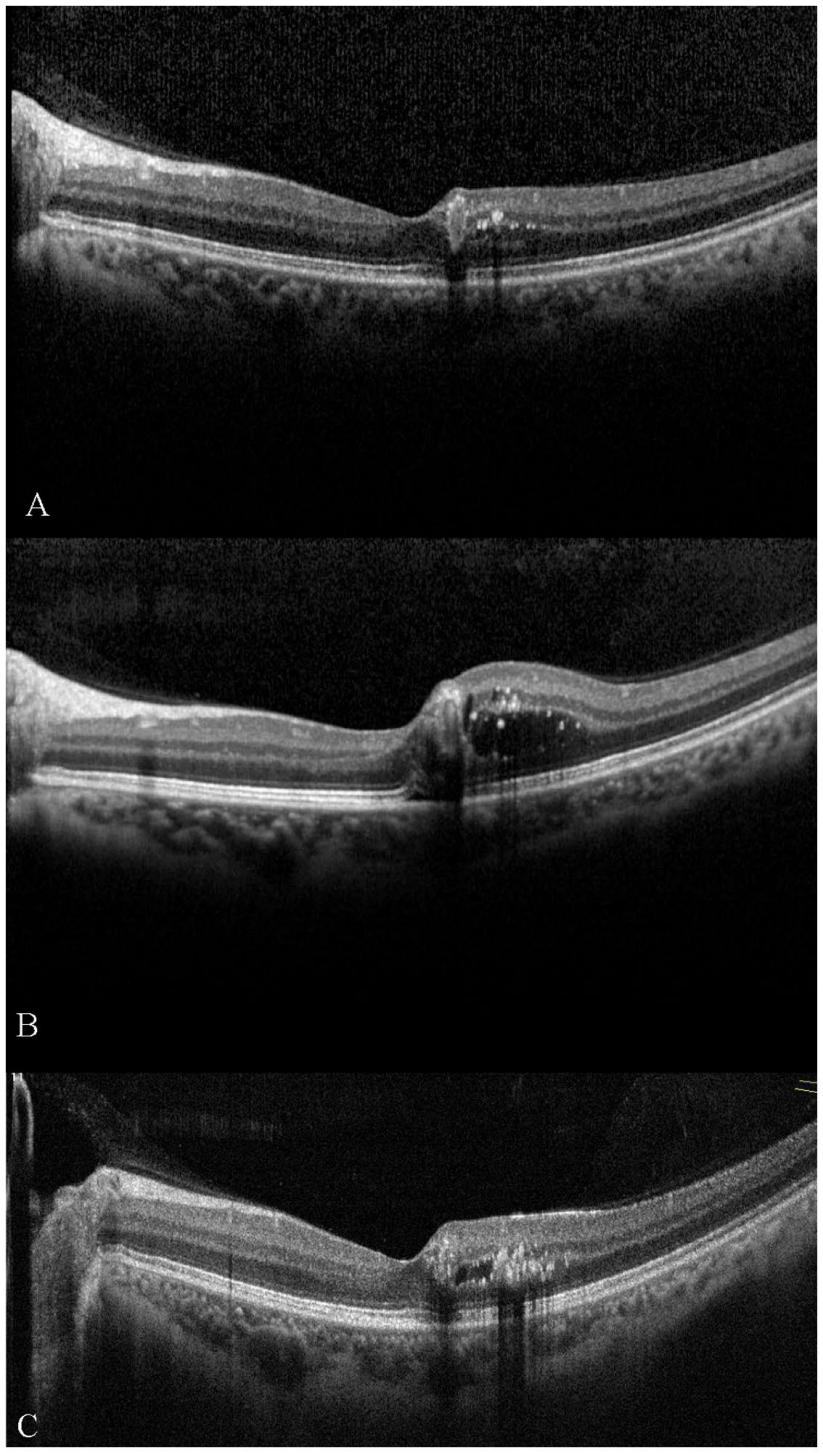
One month after intravitreal injection of ranibizumab into the left eye (19 December 2023), OCT revealed no significant change in the circular cystic lesion adjacent to the macular fovea. However, cystic interlaminar edema surrounding the lesion showed reduced volume compared to previous findings **(A)**. OCT performed 6 months after the intravitreal injection of ranibizumab into the left eye (27 June 2024, prior to menstruation). The circular cystic lesion adjacent to the fovea shows no significant change, with cystic interlaminar edema reappearing around the lesion **(B)**. OCT performed 6 months after the intravitreal injection of ranibizumab into the left eye (3 July 2024, post-menstrual period) demonstrated that the paracentral macular cystoid lesion remained unchanged. Peripheral to the lesion, cystoid interlaminar retinal edema is markedly reduced compared to previous findings **(C)**.

**Figure 3:**
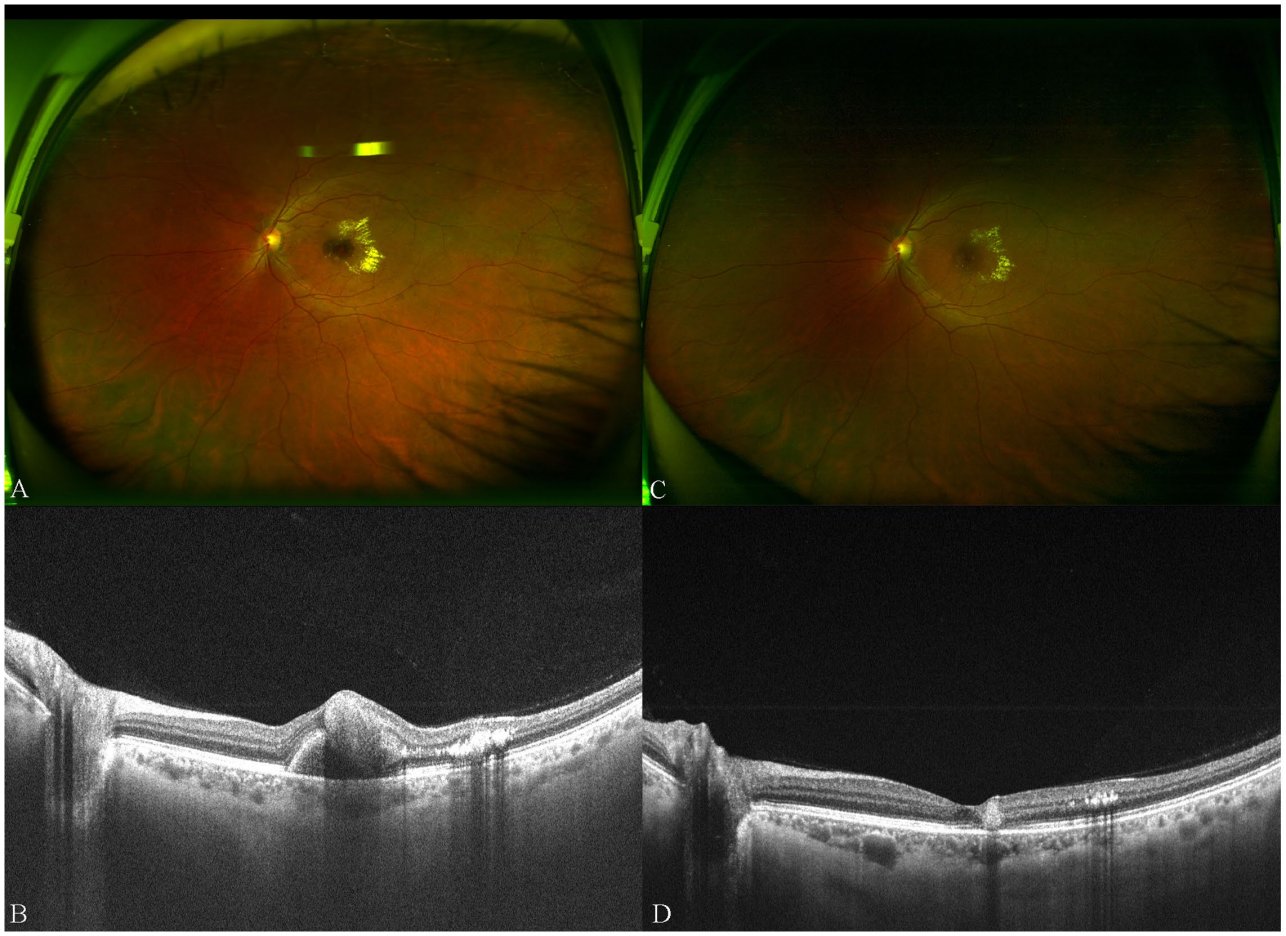
Fundus image of the patient 19 months after the intravitreal injection of ranibizumab (25 July 2025) showed patchy hemorrhage in the left macular area, with a continued increase in surrounding yellowish-white hard exudates compared to previous findings **(A)**. OCT revealed a localized, dome-shaped area of moderately to highly reflective signal in the macular fovea and beneath it, originating from the neuroepithelial layers. The lesion appeared dense with uneven reflectivity, obscuring underlying signals. Peripherally, punctate areas of high reflectivity were visible. No circular cystic lesions were observed **(B)**. One month after the fifth intravitreal injection of ranibizumab into the left eye (26 November 2025), fundus images revealed significant resolution of hemorrhage in the macular region and surrounding yellowish-white exudates compared to previous findings **(C)**. OCT images demonstrated punctate and patchy medium-intensity reflex signals visible between the neuroepithelial layers, with localized elevation showing marked regression compared to prior observations **(D)**.

**Figure 4:**
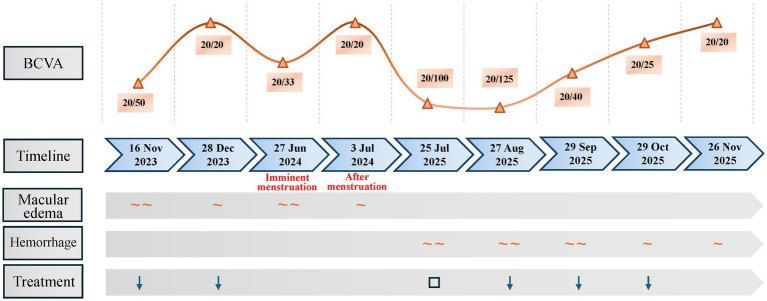
Timeline of the case. The timeline illustrates the clinical course. BCVA is plotted as a line with triangles denoting measurement time points. Macular edema and hemorrhage events are jointly represented by wavy lines, where an increasing number of wavy lines indicates greater severity. Treatment interventions are indicated by arrows (intravitreal anti-VEGF therapy) and boxes (oral medication).

The patient initially presented with macular edema in the left eye, accompanied by a marked decline in visual acuity. Previous studies have reported that intravitreal anti-vascular endothelial growth factor (anti-VEGF) therapy or retinal laser photocoagulation may effectively induce lesion regression (2–10). However, given the closeness of the lesion to the fovea centralis, laser photocoagulation posed a substantial risk of iatrogenic foveal damage and potential visual impairment. Therefore, following a comprehensive discussion with the patient and the acquisition of written informed consent, an intravitreal injection of ranibizumab was administered to the left eye on 19 November 2023. At the 1-month follow-up, the best-corrected visual acuity (BCVA) of the left eye had improved to 20/20. OCT demonstrated no significant changes in the paracentral macular cystoid lesion, whereas the surrounding cystoid interlamellar edema showed partial resolution (Figure 2A). A second intravitreal ranibizumab injection was administered on 28 December 2023. Postoperatively, the patient reported subjective symptomatic improvement but failed to attend the scheduled follow-up visits. On 27 June 2024, the patient returned with a recurrent visual decline in the left eye. The BCVA was 20/33. OCT revealed no significant changes in the circular cystic lesion adjacent to the fovea centralis. However, recurrent cystoid macular edema was observed in the perilesional region (Figure 2B). The patient reported imminent menstruation. After discussion, a follow-up was scheduled after the completion of menstruation to evaluate the necessity of a second intravitreal ranibizumab injection. On 3 July 2024, the patient returned for evaluation after menstruation and reported spontaneous visual improvement. She noted that during the preceding year, her left eye vision exhibited cyclical fluctuations correlated with her menstrual cycle, characterized by deterioration before menstruation and subsequent spontaneous recovery without intervention. At this visit, the BCVA had recovered to 20/20. OCT demonstrated marked resolution of the retinal interlamellar cystoid edema, whereas the circular cystic lesion adjacent to the fovea remained unchanged (Figure 2C). Given the satisfactory visual function, continued clinical observation was recommended. On 25 July 2025, the patient presented with a 13-day history of visual obstruction in the left eye. The BCVA had declined to 20/100 and was not correctable (−0.25 × 177). Fundus examination revealed clear optic disc margins with normal coloration, patchy macular hemorrhage, and increased surrounding yellow-white hard exudates compared to previous findings (Figure 3A). OCT demonstrated clumped moderate-to-high reflectivity within the neurosensory retina, dense hyperreflective foci in the parafoveal outer plexiform and inner nuclear layers with posterior shadowing of the underlying structures, a subfoveal dome-shaped neurosensory detachment, and no obvious abnormalities were observed in the retinal pigment epithelium (RPE) (Figure 3B). Considering the short duration and macular location of the hemorrhage, conservative management was initially adopted. The patient was prescribed oral He Xue Ming Mu Pian. At the 1-month follow-up on 21 August 2025, a subsequent FFA revealed blocked fluorescence in the macular area during the early phase. As the angiogram progressed, fluorescein leakage from the vessel walls of small inferotemporal veins in the macula and mild hyperfluorescence at the tumefaction-like lesions were observed. In the late phase, the fluorescence gradually faded. On 27 August 2025, the BCVA was 20/125, indicating a suboptimal response to medical therapy. Therefore, a third intravitreal injection of Ranibizumab was administered. Subsequent follow-up revealed progressive visual improvement. On 29 September 2025, the BCVA improved to 20/40, and a fourth intravitreal Ranibizumab injection was administered, and on 29 October 2025, the BCVA further improved to 20/25, which was followed by a fifth intravitreal injection. During the final follow-up on 26 November 2025, the BCVA had recovered to 20/20. Fundus examination images revealed significant resolution of the hemorrhage in the macular region and surrounding yellowish-white exudates compared to previous findings (Figure 3C). OCT revealed improvements in foveal contour asymmetry, attenuation of heterogeneous neuroepithelial reflectivity, reduction in punctate and clustered medium-intensity signals, and regression of localized retinal elevation (Figure 3D). The clinical timeline of this case is illustrated in Figure 4. Given the significant absorption of the macular hemorrhage and sustained visual recovery, the patient remains under close clinical observation.

## 3 Discussion

PEVAC is a rare macular disorder first described by Querques et al. (1) in 2011. PEVAC presents clinically as isolated, circular aneurysmal lesions surrounding the fovea centralis, typically occurring in otherwise healthy patients without retinal inflammation or vascular abnormalities. In 2020, Sacconi et al. (11) identified a potential non-exudative stage of PEVAC and proposed classifying the condition into exudative and non-exudative perifoveal vascular anomalous complex based on the presence or absence of exudative changes.

During fundus examination, PEVAC presents as an isolated aneurysmal lesion near the fovea centralis, with or without hemorrhage or hard exudates. On OCT, PEVAC appears as a circular or oval hyper-reflective lesion accompanied by hyper-reflective cystic cavity, potentially surrounded by retinal interlamellar cysts (12, 13). OCTA reveals a circular tubular structure with a highly reflective wall adjacent to the fovea, containing blood flow signals. This may manifest as abnormal blood flow signals in the superficial and/or deep capillaries, with no blood flow signals observed in the avascular layer of the retina. During FFA, the early phase reveals a well-defined hyperfluorescent lesion adjacent to the fovea with minimal or no late-phase fluorescein leakage. ICGA shows a well-defined hyperfluorescent lesion at the foveal margin with no significant late-phase leakage. No other retinal or choroidal vascular abnormalities were observed during examination (14, 15).

The pathogenesis of PEVAC remains unclear. Sacconi et al. (11) suggested that it may be associated with retinal endothelial cell degeneration. Spaide et al. (2) proposed that the increased expression of matrix metalloproteinase-9, which degrades basement membrane proteins, leads to a reduction in pericytes. This imbalance between decreased vascular wall strength and increased vascular wall tension may trigger the formation of aneurysmal lesions. Furthermore, Müller cells may play a role in lesion formation because of their functions in maintaining the blood–retinal barrier and regulating the extracellular space volume. This could also explain the concurrent occurrence of PEVAC with macular holes reported by Siedleck et al. (16).

As the pathophysiological mechanisms are yet to be fully understood, the optimal therapeutic approach for PEVAC remains undetermined. Given its relatively slow progression, some researchers recommend initial clinical observation, noting that spontaneous remission has been observed in some cases during follow-up (12). Various treatment approaches have been proposed, including anti-VEGF intravitreal injections (1, 3–7, 13, 17, 18), retinal laser photocoagulation, and topical non-steroidal anti-inflammatory drugs. Some studies have demonstrated that anti-VEGF agents alone (4–6), in combination with triamcinolone (7), or with retinal laser therapy (3) can lead to disease remission. In the present case, anti-VEGF therapy administered early during disease detection led to an initial improvement. However, lesion rupture and hemorrhage occurred as the disease progressed. Subsequent anti-VEGF treatment after the hemorrhage resulted in better control than previously. This pattern of disease progression and the corresponding treatment approach have not been described in reported cases of PEVAC. Furthermore, this study proposes that anti-VEGF agents facilitate the absorption of early cystoid interlaminar edema surrounding the lesions and late-stage hemorrhage from ruptured lesions, thereby alleviating symptoms. However, they fail to halt disease progression. In the early phase of lesional hemorrhage, the orally administered oral He Xue Ming Mu Pian facilitate the absorption of the fundus hemorrhage. He Xue Ming Mu Pian is primarily indicated for fundus hemorrhagic diseases and is prepared from Chinese herbal medicines under the guiding principle of syndrome differentiation and treatment in traditional Chinese medicine, and their formulation is informed by modern pharmacological research on Chinese materia medica (19). Che Xinhua (20) reported that He Xue Ming Mu Pian, when used to treat vitreous hemorrhage secondary to various retinal vascular diseases, rapidly arrests bleeding, promotes the absorption of blood stasis, reduces the risk of recurrent bleeding, and contributes to the prevention of severe complications such as retinal detachment secondary to fibrosis. Additionally, He Xue Ming Mu Pian has been reported that when used in combination with other therapeutic modalities for patients with wet age-related macular degeneration (wAMD) accompanied by macular hemorrhage, this drug facilitates the absorption of retinal hemorrhages by modulating ocular blood flow (21, 22). Following this treatment, the patient showed no significant improvement and ultimately underwent anti-VEGF therapy. Various types, patterns, and power levels of retinal laser therapy have been clinically applied (2, 4, 8–10). However, because of the proximity of PEVAC lesions to the fovea centralis and the potential for laser treatment to cause retinal tissue damage, including visible retinal scarring, laser scar enlargement, secondary hemorrhage, or secondary choroidal neovascularization leading to further macular dysfunction, there remains a risk of permanent paracentral scotoma formation (23). The use of topical non-steroidal anti-inflammatory drugs for PEVAC has been reported in a few studies; however, their efficacy requires further investigation (24).

Following the detection of macular hemorrhage, the following differential diagnosis was performed. First, it is necessary to differentiate this condition from retinal arterial macroaneurysm (RAM). RAM is a clinically acquired retinal vascular disorder that typically manifests as a localized cystic or fusiform aneurysmal dilation of a blood vessel, most commonly involving the third-order branches of the retinal artery (25–28). The condition typically affects one eye, with lesions often located on the temporal side, and is most common in elderly women. This condition is commonly associated with hypertension and atherosclerosis (29, 30). The postulated mechanism is that chronic hypertension and aging induce degeneration of the arterial wall, thereby resulting in localized dilation (27). In cases of non-hemorrhagic RAM, FFA may reveal a brightly fluorescent spot at the lesion site with well-defined borders. If the lesion ruptures and results in minor hemorrhage, a corresponding area of fluorescence blockage may be observed. In the late phase of angiography, varying degrees of fluorescein leakage or staining may occur at the lesion site, leading to irregular staining patterns (31). However, when severe hemorrhage obscures the lesion, adequate visualization becomes difficult, and a definitive diagnosis cannot be established. OCTA reveals hyperreflective signals originating from the RAM within the superficial retinal layers, and these signals are continuous with the retinal arteries (32). Although this patient presented with signs of macular hemorrhage, she was a 44-year-old woman with no history of hypertension, and no signs of arteriosclerosis were observed in her fundus vessels. Moreover, the lesion was located in close proximity to the macula, and no branch retinal arteries were visualized in that region. Accordingly, a diagnosis of RAM could be excluded. OCT imaging performed following the patient’s hemorrhage revealed that the bleeding was localized to the retinal neuroepithelium and the subneuroepithelial space, while the RPE appeared structurally intact. Given that the patient was a 44-year-old Chinese woman, conditions including polypoidal choroidal vasculopathy, wAMD, and choroidal neovascularization could be excluded. Valsalva retinopathy was first described by Duane (33). Patients often report a clear Valsalva maneuver prior to symptom onset, including forceful coughing, vomiting, sneezing, straining during defecation, heavy lifting, moving heavy objects, or engaging in strenuous physical activities such as pull-ups and push-ups. These actions induce a rapid and transient elevation of intrathoracic and intraabdominal pressure, leading to a sudden increase in intraocular venous pressure. Consequently, rupture of superficial capillaries around the macula occurs, resulting in preretinal hemorrhage; when hemorrhage is extensive, it may severely compromise visual acuity. In the present case, the patient denied any history of the aforementioned conditions, and the hemorrhage was localized to the retinal neuroepithelial and subneuroepithelial layers rather than the preretinal space. Therefore, a diagnosis of Valsalva retinopathy may be excluded.

Regarding the transient visual changes occurring with menstruation described in the case report, this study conducted a literature search but found no related descriptions or analyses in previously reported cases. Estrogen and progesterone, the two primary hormones associated with the menstrual cycle, can affect the blood vessels in the body. One shared effect of estrogen and progesterone is increased vasodilation (34, 35). Estradiol, the most potent estrogen in humans, enhances *β*-adrenergic receptor-mediated vasodilation, thereby counteracting *α*-adrenergic-mediated vasoconstriction (36), and influences nitric oxide (NO) production and activity (37). Progesterone enhances NO synthesis through both transcriptional and non-transcriptional pathways at the cellular level, potentially affecting the microvasculature but not the macrovasculature *in vivo* (38, 39). Concurrently, sympathetic nervous system activation may compensate for NO effects, thereby maintaining vascular pressure equilibrium (40). Karadeniz et al. (41) studied 23 healthy women using color Doppler ultrasound to investigate posterior ocular circulation during their normal menstrual cycles and found that hemodynamic parameters remained stable throughout the menstrual cycle. Guo et al. (42) recruited 62 women with an average age of 27 years and used OCTA to study vascular changes during the follicular, ovulation, and luteal phases. They found no significant differences in the vascular density within the superficial capillary plexus of the retina. However, compared to the follicular or luteal phase, the vascular density in the deep capillary plexus of the nasal and inferior regions was significantly reduced during ovulation, indicating fluctuations in the microvascular system throughout the menstrual cycle. Another study found no significant differences in the vascular density of the superficial or deep capillary plexuses during the follicular, ovulation, or mid-luteal phases without any intervention (43). As a vascular anomaly complex, the association between PEVAC and accompanying peripapillary cystoid edema, exudation, or even hemorrhage from the lesion itself with the menstrual cycle remains unclear. This phenomenon was derived from patient’s subjective experiences, and due to individual variations, this study observed changes around the time of menstruation on only one occasion during the course of this interesting phenomenon. This study was unable to conduct further observations or research in a timely manner, including continuous OCT measurements synchronized with the phases of the menstrual cycle and hormone level testing. This underscores the limitations of this study. During the literature review, this study have not, to date, identified any studies on similar phenomena. Furthermore, this study recommends conducting an observational study to evaluate the effect of the menstrual cycle on disease progression in premenopausal adult women with PEVAC. We will continue to follow this patient to monitor changes in her ocular status, and hope that the findings will provide new perspectives for research regarding the disease pathogenesis.

## 4 Conclusion

The case presented underscores that PEVAC may lead to the rupture and hemorrhage of secondary lesions. Gradual improvement in the patient’s vision was achieved through intravitreal anti-VEGF drug injections. This study observed that the menstrual cycle in women may be associated with disease progression. However, the relationship between the menstrual cycle and disease progression remains unclear.

## Data Availability

The raw data supporting the conclusions of this article will be made available by the authors, without undue reservation.
